# Electrospun Polymer Fibers for Electronic Applications

**DOI:** 10.3390/ma7020906

**Published:** 2014-01-28

**Authors:** Alessandro Luzio, Eleonora Valeria Canesi, Chiara Bertarelli, Mario Caironi

**Affiliations:** 1 Center for Nano Science and Technology @PoliMi, Istituto Italiano di Tecnologia, Via Pascoli 70/3, 20133 Milano, Italy; E-Mails: eleonora.canesi@iit.it (E.V.C.), mario.caironi@iit.it (M.C.); 2 Dipartimento di Chimica, Materiali e Ing. Chimica “G. Natta”, Politecnico di Milano, Piazza L. Da Vinci 32, 20133 Milano, Italy

**Keywords:** electrospinning, organic semiconductors, conjugated polymers, conductive polymers, Organic Field Effect Transistors (OTFTs)

## Abstract

Nano- and micro- fibers of conjugated polymer semiconductors are particularly interesting both for applications and for fundamental research. They allow an investigation into how electronic properties are influenced by size confinement and chain orientation within microstructures that are not readily accessible within thin films. Moreover, they open the way to many applications in organic electronics, optoelectronics and sensing. Electro-spinning, the technique subject of this review, is a simple method to effectively form and control conjugated polymer fibers. We provide the basics of the technique and its recent advancements for the formation of highly conducting and high mobility polymer fibers towards their adoption in electronic applications.

## 1. Introduction

Conjugated polymers offer the possibility to develop flexible and light-weight opto-electronic applications thanks to their solubility and low-temperature processing [1–3]. Both conductors [4–6] and semiconductors [7] are available, enabling in principle an all-organic electronics when combined with more traditional insulating plastics. This technology would be therefore substantially based on carbon, an earth-abundant element. While being characterized by limited performances with respect to other inorganic technologies, recent synthetic and processing advancements clearly make conjugated polymers even stronger candidates for future large-area, flexible electronics. Polymer conductors with conductivity values of a few 10^3^ S/cm have been demonstrated [8], and semiconductors with both p- and n-type carriers mobility exceeding 10 cm^2^/Vs, with improved ambient stability are now available [9,10].

The development of suitable deposition techniques is crucial to fully exploit the potentiality of conjugated polymers. Besides the development of printing tools for the controlled patterning of polymer films, the possibility to deposit them in the form of micro- and nano-fibers is a very attracting and emerging option. Extended wires of polymers offer unique systems characterized by an improved mechanical strength, an increased surface-to-volume ratio, and quasi 1-D dimensionality in the case of nano-fibers, where studying charge transport in a confined system. Various electronic functionalities can be implemented into different fibers, enabling to fabricate different devices and components: these include light-emitting diodes, photovoltaics [11] and field-effect transistors, besides a broad series of sensors [12–15] and other photonic components such as optically pumped lasers, and waveguides. We address the interested reader to a recent review by Lee and co-workers [16] covering most of these applications. One of the possible tangible outcomes of functional fibers is the development of “smart textiles”, where fabrics are equipped with integrated electronic devices during their production, enabling a ubiquitous application of wearable electronics. While this perspective may still appear visionary, and without doubt several issues have to be solved, with this contribution we aim at providing a clear reference for the state of the art and at clarifying which approaches can be more promising for the development of this field.

Here we specifically focus on electrospinning, which is one of the best known methods to produce polymer fibers and stands out among others due to its simplicity, which enables the continuous formation of fibers composed of a broad range of insulating, conducting and semiconducting polymers, or even multi-component fibers, with diameters ranging from a few hundreds of nanometers to several hundreds of micrometers. This simplicity has facilitated its adoption in research laboratories, conducting pioneering experiments on the formation of functional single- and multi-component fibers [17], and of corresponding opto-electronic devices.

We describe the electrospinning technique, with recent interesting developments for the control of the fiber alignment in Section 2. With respect to other contributions in the literature [16], we then focus on the materials adopted to form conducting (Section 3) and semiconducting fibers (Section 4). We have selected the most notable examples in the literature that can indicate in different ways the full potentiality of this approach. In the case of semiconductors we have focused on fibers based field-effect transistors, seen both as a way to extract charge carriers mobility and as a building block for future logic functions in smart textiles (Section 4.3). In this context we pay particular attention in describing the advantages and limits of different methodologies followed in the literature to extract mobility and threshold voltage parameters (Section 4.1). The results of a survey of conductivities and carriers mobility achieved so far in polymer fibers have been reported in Tables 1 and 2.

## 2. Electrospinning

Electrospinning is a simple, cheap and versatile technique to form polymer fibers with section in the micro- and nanoscale [18–22]. The technique exploits high electric potentials to overcome the surface tension of the fluid. Despite the fact that the first studies were patented about one century ago [23], electrospinning has gained growing industrial and academic interest since the 1990s [24].

Electrospinning requires viscoelastic properties typical of polymeric materials: it has been mostly applied to polymer solutions. Nevertheless, processing from melt has been also performed, but more challenging conditions are required. Many natural and synthetic polymers have been successfully electrospun, including conjugated polymers since the pioneering work by MacDiarmid [25].

### 2.1. Basic Aspects of the Technique

**Figure 1.**
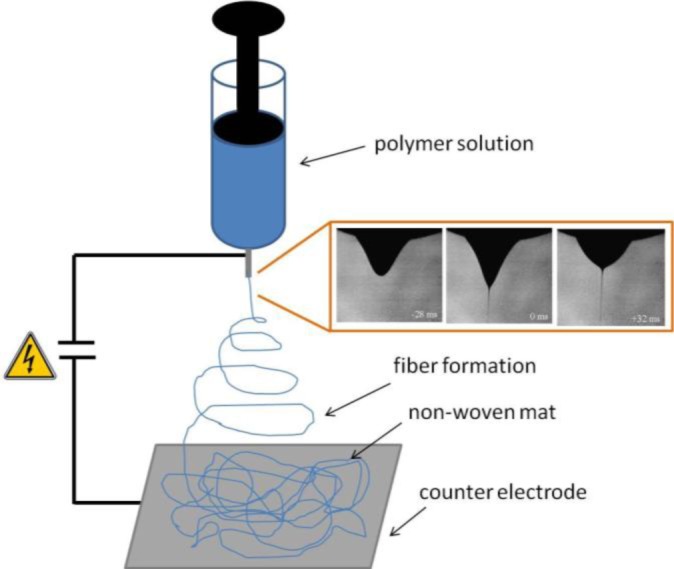
Sketch of a typical laboratory electrospinning setup; in the inset, frames from a video representing the evolution in time of the shape of a fluid drop with electrical potential. Adapted with permission from [26]. Copyright (2008) by Elsevier.

The electrospinning technique consists in the application of a high positive voltage, typically 10–30 kV, to a polymer fluid, usually contained in a syringe, with respect to a grounded collector (Figure 1). The applied voltage produces an accumulation of ions present in the fluid at the air-fluid interface, and when the electrostatic repulsion overcomes surface tension, a fluid jet erupts. The jet travels towards the region of lower potential experiencing an elongation with a strong diameter reduction during the flight [26]. The jet is characterized by different regions, namely: (i) a droplet at the tip of the syringe where the polymer solution is contained, which assumes a conical shape (Taylor cone) due to the balance between electrostatic repulsion and cohesion forces [27]; (ii) a stable straight region close to the syringe tip, with length in the order of 1 cm and (iii) a region of unstable whipping (or bending) motion, with the jet moving laterally and forming a series of coils, the envelope of which draws a cone opening towards the counter electrode [28], where fibers are collected as a nonwoven mat. The jet under the electric field is driven with acceleration of orders of magnitude larger than that provided by gravitation, thus gravitational forces do not play a significant role [29].

**Figure 2.**
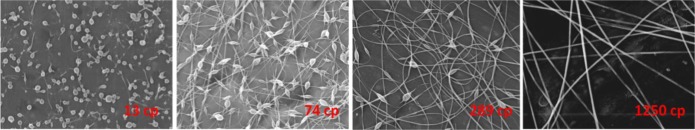
Evolution of the morphology of polymer fibers upon increase of solution viscosity. SEM images of poly(ethyleneoxide) fibers (each image shows a 20 μm wide area), together with viscosity value (in red). Left: polymer drops and fibers with spherical beads. From left to right, as the viscosity progressively increases, the average distance between beads on the fibers becomes larger and the shape of the beads changes from spherical to spindle-like, till reaching uniform fibers. Adapted with permission from [30]. Copyright (1999) by Elsevier.

The viscoelastic properties of the polymer solution represent a key factor in the electrospinning process, since a critical amount of chain entanglements is needed for fiber formation. Below this critical value, which differs for each system composition, the voltage applied results in electrospraying or in the formation of beaded fibers due to a capillary wave breakup (Rayleigh instability) [30]. The more viscous the system, the less defective the fibers; however, too high viscosity turns into high cohesiveness of the solution and may cause flow instability (Figure 2).

The uniformity of the fibers, the absence of defects, the average diameter and their distribution width are all characteristics which influence the final quality of the deposited fiber mat. Incidentally, the stability of the spinning process, which gives homogeneous fibers with narrow distribution of diameters, can be monitored during the process through the current flowing between the electrodes, which stops oscillating when the deposition becomes stable [31].

### 2.2. Control of Fibers Formation

The actual fibers formation and their morphology depend on many processing parameters, which are classified as setup parameters (*i.e.*, applied voltage, volume feed rate, tip-to-collector distance, needle inner diameter, collector type), solution parameters (*i.e.*, polymer concentration and molecular weight, solvent electrical conductivity and boiling point, solution viscosity, surface tension) and environmental conditions (*i.e.*, temperature, pressure and relative humidity). They are usually highly interrelated, nevertheless, some have a prevailing effect with respect to others.

As rheological properties give access to a stable spinning process, it is clear that solution parameters like polymer concentration and molecular weight, as well as polidispersity play a relevant role [32]. As a reference, solutions with a concentration of polymer of some % in weight are used in electrospinning. Tan *et al.* [33] demonstrated that these factors, together with the electrical conductivity of solvents, have a “primary effect” with respect to others (voltage and feed rate) which are classified as “secondary parameters”. A theoretical study has supported this conclusion [34]. However, as a general statement, empirical data still retain a fundamental role in determining the optimal conditions to gain high quality electrospun fibers [35,36].

It is not trivial to predict the influence of the solvent on the resulting fiber morphology, since it depends on the combination of its boiling point, vapor pressure, viscosity, surface tension and conductivity; the situation is even more complex when solvent mixtures are used or the feed solution is loaded with additives, such as salts to increase the solution conductivity.

All parameters in the spinning process have to be set within a suitable range of values: just to mention an example related to the feed rate, that is usually in the 0.01–10 mL/h range, low feed rates make the spin process discontinuous while at high feed rates the voltage is not sufficient to generate the jet from the continuously forming droplet [37]. Moreover, although too low bias does not allow an effective drop charging, too high values may cause multiple jets. Depending on the specific polymer-to-solvent system the applied voltage has also opposite effects, since it acts both on the mass of polymer fed out from the needle and on the jet elongation driven by the electric field [38]. Finally, ambient parameters have been found to affect the surface morphology, and relative humidity specifically plays a role in affording porous fibers. For a comprehensive overview on the effect of the different parameters on the fiber morphology, we address the interested reader to the work of Reneker and co-workers [26–30,34,39].

### 2.3. Alignment of Fibers

The collection of fibers aligned along a preferential direction is a very relevant aspect to be considered for various applications: opto-electronic devices could benefit from an ordered deposition of conducting/semiconducting fibers as a mean to access and control anisotropic electronic properties, while for bio-related applications it has widely demonstrated that scaffolds with aligned fibers favor cells growth along a preferential direction [20]. Moreover, the access to samples with aligned fibers simplifies the investigation of the polymer chains orientation within them: indeed, the significant jet elongation during the electrospinning process can drive the orientation of the polymer backbones in the direction of the fiber axis [40–44]. Polarized spectroscopy techniques, such as infrared and Raman spectroscopy [42,45–47], UV-vis and photoluminescence [48], as well as birefringence in optical microscopy, can be conveniently used with this aim. Chain orientation does not usually correspond to an increased crystallinity, due to the fast solidification of the jet with respect to the crystal nucleation and growth processes [49].

**Figure 3.**
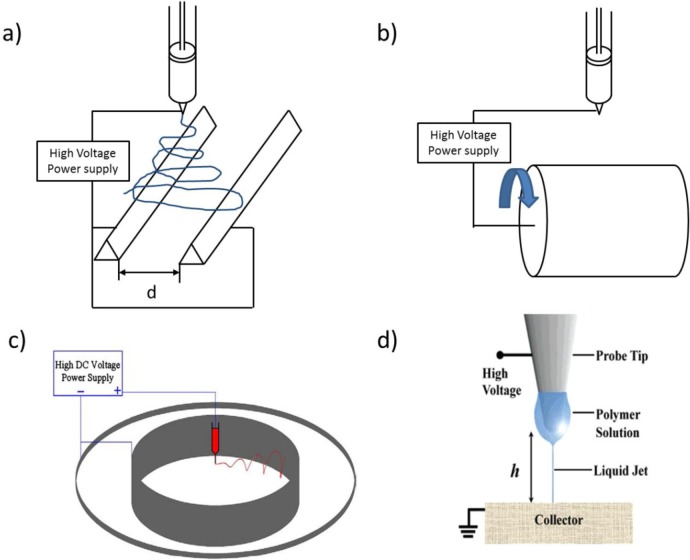
Examples of methods to induce fibers alignment: (**a**) parallel electrodes; (**b**) rotating collector; (**c**) rotating jet method and (**d**) near field electrospinning. Adapted with permission from [40]. Copyright (2011) by Elsevier. Adapted with permission from [56]. Copyright (2006) by American Chemical Society.

Several strategies have been proposed to induce a preferential fibers alignment (Figure 3). The parallel electrode collector principle, where fibers lie in the gap to minimize the torque generated by the electrostatic forces, is the simplest technique to gain fibers orientation; as expected, fibers alignment is significantly improved while decreasing the gap width [50]. However, this method suffers for partial misalignment of the above fiber layers [51]. Mechanical methods based on rotating collectors [52], or grounded frames rapidly oscillating within the jet, or also the use of charged collectors, allow overcoming these issues. For mechanical methods, the relation between the linear speed of the dynamic collector surface and the jet deposition (which falls in the 2–200 m/s range) is of critical importance. The geometry of the system (*i.e.*, “rotating jet method” using a hollow metallic cylinder) [40] as well as the tip-to-collector distance are also relevant [53] for an effective control over fibers alignment. The electrospun jet from polymer solutions containing magnetic nanoparticles can also be directed by applying magnetic fields [54], or employing auxiliary electrodes, which modify the electostatic field [55] (*i.e.*, an electrically earthed collector to gather the nanofibers is used) [51].

A powerful strategy to deposit well aligned and controlled fibers is to collect them at a short distance from the syringe orifice, before the bending instability occurs; this method is named “near-field electrospinning” or “precision electrospinning” [56–58]. The distance between spinneret and collector, that ranges between a few millimeters and a few centimeters, depends on different parameters, e.g., the flow rate (*Q*); the surface charge (σ); the conductivity (*k* is the dimensionless conductivity of the solution); the applied electric field (*E*); the current passing through the jet (*I*); the liquid density (ρ); the needle radius (*r*_0_). In the simplest configuration, namely when auxiliary electrodes are not present, the following equation that relates all these parameters has been proposed to predict the length of the stable jet (*L*) [59]:

$$L=\frac{4kQ^{3}}{\pi \rho^{2}I^{2}}\left[{\left(\frac{\pi k\rho E}{2\sigma Q}\right)}^{2/3}-r_{0}^{-2}\right]$$ (1)

Such length increases with higher evaporation rate solvents or more viscous solutions, making near field electrospinning particularly suitable for melt electrospinning [60]. Also, alternated current and higher applied voltage have a contribution in the same direction, while lower voltages allow a more precise control over the spinning direction thanks to a lower flux acceleration [28]. Near field electrospinning presents similarities with inkjet printing: a modified printing apparatus called “high-resolution electrohydrodynamic jet printing” [61] has been demonstrated also for the production of semiconducting nanowire arrays [62]. In case of precision electrospinning, the jet is continuous, while in case of printing lines are formed by the deposition of single droplets that coalesce onto the substrate. Although scanning collectors are usually employed, scanning tips have also been proposed to control the fiber deposition and alignment [63].

In recent years, the basic electrospinning setup has been modified to enhance process stability and fibers productivity or to produce fiber yarns, self-standing meshes or three-dimensional scaffolds. We refer to recent reviews for a comprehensive overview on the topic [22,51,64,65].

### 2.4. Electrospinning of Conjugated Polymers

**Figure 4.**
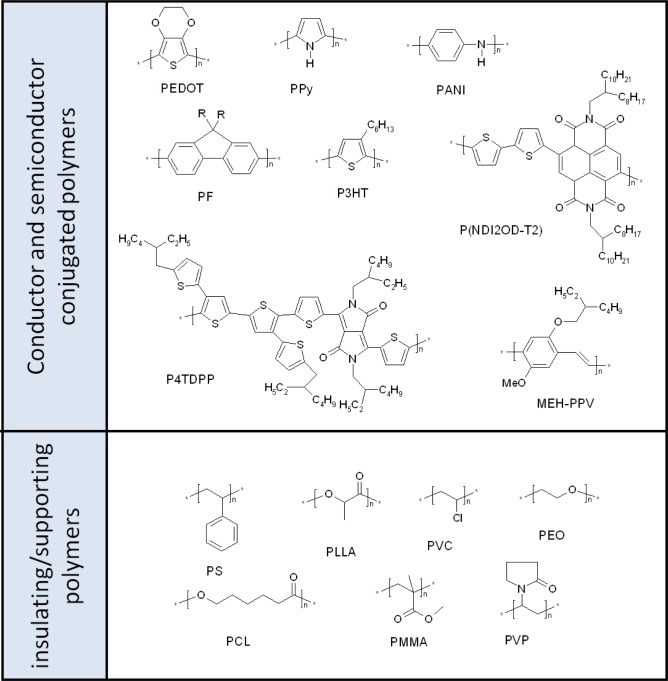
Chemical structures of electrospun conjugated and insulating/supporting polymers.

Within this broad scenario, electrospinning of conjugated polymers (in Figure 4 the most representative conjugated polymers processed by electrospinning are reported) provides additional requirements and challenges. Indeed, conjugated polymers are characterized by a limited solubility, by a relatively low average molecular weight and by a more rigid backbone: all these characteristics limit the number of entanglements that assist the fibers formation during electrospinning, preventing the jet breaking under the elongation strength. Moreover, though it has been shown that not-completely-good solvents may favor the spinnability [66], a good polymer solubility is required to reach the minimum solution viscosity for fiber formation [32].

Regarding solubility and molecular weight, poly-p-phenylenes (PPVs) are an exception and they have been electrospun as pure materials giving defect-free fibers [67,68]. In most cases instead, a more spinnable compound, is added to the feed solution to assist the fibers formation (Figure 4). In this way multicomponent structures are obtained, where the conjugated polymer has the role to impart an optical or electrical functionality. Well soluble polymers with high molecular weight are preferred as supporting material: poly(ethyleneoxide) (PEO) [69,70], poly(methylmethacrylate) (PMMA) [71], polystyrene (PS), [72] poly(vinylpyrrolidone) (PVP) and [73] poly(ε-caprolactone) (PCL) have been extensively used with this regard [74]. A proper choice of the supporting polymer allows its selective rinsing aimed at obtaining fibers of the pure conjugated polymer [75–78].

Despite the reports that functional properties can be retained or even enhanced in a multicomponent fiber with respect to a polymer thin film [78], the intermixing of the two materials may prevent the continuity of one of the two phases negatively affecting the fiber electrical properties [69,74]. Interestingly, blend solutions have shown to afford concentric fibers under specific conditions, where the more viscous polymer spontaneously locates in the inner of the fiber. This effect, which has been widely investigated for traditional polymers [79], has been also obtained in case of conjugated polymers such as polyfluorene (PF) [50,80] or polyaniline [81]: in both cases, the conjugated moiety turns out to be located in the fiber core, mainly owing to its lower solubility.

Two methods are available to reproducibly obtain two-component fibers with a concentric distribution of the two compounds. The first one makes use of coaxial capillary spinnerets. The core usually retains the peculiar electrical or optical properties while a polymer with proper viscoelastic characteristics is used to form the sheath. Coaxial spinning by means of concentric needles has been firstly introduced in 2003 [82]. A demanding balance of all the parameters is strictly required to obtained core-sheath structures and avoid formation of eccentric fibers or mixing of the two phases [83]. To gain continuous domains of the two components, immiscible systems are generally required; suitably acting on miscibility conditions, bicomponent fibers with different morphologies, such as fibers containing inclusions [84] or horizontal cylinder segments [85], can be accessed by means of two capillary spinnerets. To overcome the issue of miscibility of the two feed solutions, a multifluidic approach with three concentric needles has been proposed, the middle fluid acting as spacer [86]. Core-sheath double spinneret setup has been also employed for gas-assisted electrospinning: here, single component fibers are obtained, while the external jacked is fluxed with a gas that protects, stretches and may also warm up the inner solution [87,88]. Solvent-assisted electrospinning, in which a solvent sheath protect and support the jet coming from the inner needle, is also possible [74].

The second method to afford concentric two-component fibers is based on template synthesis, where electrospun supporting fibers, typically composed of insulating materials, are subsequently coated (e.g., by thermal evaporation, *in-situ* polymerization, *etc*.) with a functional material as the shell. In case of concentric structures, selective rinsing of the supporting polymer may also afford hollow fibers: tubes of conjugated polymers have been developed with this method [89,90].

The three different methods above discussed, namely electrospinning of blends, surface deposition or coaxial spinning, present different constraints and advantages, which govern their suitability with respect to the properties of the compounds of interest. As a general guideline, direct spinning of polymer blends provides a clear advantage in terms of setup simplicity, and relax the requirement of having immiscible solutions for the core and the sheath. However, it poses different constraints, especially if a concentric structure is addressed. Indeed, for blend systems, kinetics more than thermodynamics was found to govern the formation of core-sheath phase separation, thus the polymer molecular mobility results to be the key parameter [79]. Template synthesis is more often used for conjugated polymers having low solubility, but that can be easily obtained by oxidative polymerization. Examples of polymerization onto fibers surface include template synthesis of polypyrrole (PPy) [91,92], poly(3,4-ethylenedioxythiophene) (PEDOT) [76,93,94] and polyaniline (PANI) [95].

## 3. Electrically Conductive Polymer Fibers

In the following, we will analyze in detail the literature concerning fibers produced through direct spinning of the conjugated polymer (an essay is reported in Table 1). However, since a significant amount of literature deals with the post-deposition of conducting polymers onto preformed fibers, a paragraph is dedicated to this approach. The main application of conducting fibers is in the sensors field, where an extended surface area is a clear advantage [96,97]. We just mention that good electrical properties may also be afforded by calcination or carbonization of electrospun polymers by thermal treatments to give carbon nanofibers and nanotubules [98,99].

### 3.1. Measurement of the Conductivity of Polymer Fibers

Electrodes pre-patterned onto the substrate or evaporated on the top of the electrospun material have been employed for the electrical characterization of single fibers and non-woven mats. Two-probe or four-probe measurements were alternatively carried out to extract conductivity values from *I*-*V* curves, differently accounting for the drop of potential occurring at the contact between the electrodes and the fiber, due to contact resistance effects; a two-probe measurement results in *I*-*V* characteristics in which the potential drop between the two probes only can be measured, which just in case of negligible contact resistance can be approximated to the drop of potential within the semiconductor. However, in case of highly conductive fibers, a contact limited behavior may be observed, so that a four-probe method must be adopted: two outer contacts are employed for the application of an external voltage, producing an electrical current (*I*) flowing through the conductor; the resultant potential drop (*V*) is measured between two other high-impedance inner contacts. Another conductivity extraction method also employing four probes is the so called van der Pauw’s four-probe method, which is particularly useful in case of non-woven mats on a substrate which can be approximated with a bi-dimensional film with almost uniform thickness and almost simply connected surface [100,101]. Nevertheless, morphological and geometrical analysis are generally required to obtain the real thickness and porosity of the mat under investigation needed to properly extract conductivity values with any of the procedure here mentioned.

In 2012, Zhang and Rutledge [102] observed that a fine electrical contact can be obtained by hot-pressure of the fibers onto the electrodes. They also estimated the contact resistance, by measuring the total resistance of fibers on interdigitated electrodes with variable finger spacing and by extrapolating the resistance value at zero spacing.

### 3.2. Single Nozzle Spinning of Conducting Fibers: One Component Fibers

PANI is by far the most employed conjugated polymer to develop conducting fibers. It has been both directly electrospun, with or without an insulating polymer which supports the fiber formation, or post-deposited onto electrospun fibers. A pioneering work dated 2000 [103] reported PANI fibers with high conductivity (150 S/cm), obtained by direct spinning of PANI solutions in dichloroacetic acid to a coagulation solvent; conductivity was further enhanced to 1950 S/cm after drawing of the electrospun mat. The same method provided PANI fibers from sulfuric acid, showing different conductivity values, respectively of 1 S/cm [104] and of 0.1 S/cm [105]. This large difference in the conductivity values can be ascribed to the difference in the fiber diameters: indeed, the highest conductivities refer to fibers with 220 μm diameter [103] and 100 μm diameter (up to 52.9 S/cm) [104]. In [106] a relation between the fiber diameter and the level of doping has been suggested, confirming an increase in conductivity for thicker fibers: a higher volume to surface ratio causes a relatively slower loss of solvent by evaporation and, consequently, the fiber is more partially doped and conductive. Conversely, data shown in [104] demonstrated higher conductivity for thinner fibers, likely due to an orientation of the polymer chains within the fiber. All these values refer to single fiber (or single bundle) measurements.

Also, PPy has been electrospun without a supporting polymer matrix. For this polymer, electrical measurements have been carried out on nonwoven mats, and remarkably different values of conductivity have been reported in the literature, likely due to the characterization method employed: for example, an increase of nearly one order of magnitude was observed by compressing the mat before the measurement (10^−2^ S/cm [107] *vs.* 0.5 S/cm [100]).

### 3.3. Single Nozzle Spinning of Conducting Fibers: Multi Component Fibers

Blending the conjugated material with a polymer supporting the electrospinning process usually affords fibers of improved quality and morphology. However, the presence of an insulating matrix may affect the fibers conductivity, depending on the polymers intermixing and the continuity of the two phases.

**Figure 5.**
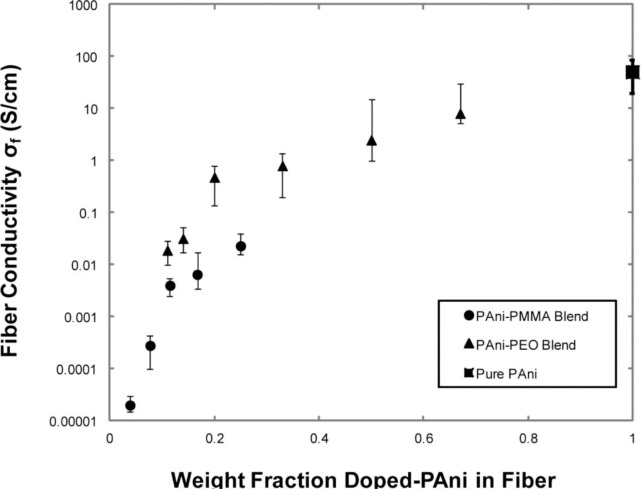
Electrical conductivity of as-electrospun polyaniline (PANI) fibers (nominally doped with an equimolar amount of D,L-camphorsulfonic acid, HCSA) as a function of the weight fraction of PANI in the blended fibers; the pure PANI fiber was obtained after dissolving the shell component (PMMA) of the core−shell fibers. Reprinted with permission from [102]. Copyright (2012) by American Chemical Society.

The literature is consistent in reporting higher conductivity values for higher conducting polymer/supporting polymer ratios. See for example the works of Chronakis *et al.* [107], Norris *et al.* [25] or the systematic study of Zhang *et al.* [102] (Figure 5), where the effect of the nature of the supporting polymer on electrical performance is also highlighted.

Fiber diameter and polymer alignment within the fiber also play a relevant role: in [105] conductivity is found to increase of more than one order of magnitude when fiber diameter is increased from 419 to 1320 nm, despite the higher content of PEO within the blend (50% *vs*. 72% respectively); this has been explained with alignment of PANI in the sample with higher PEO content. Moreover, thinner fibers (below 100 nm [108] or few hundred nanometers [69]) have found to be insulating or highly resistive, despite a low supporting polymer content: the small diameter may allow enhanced de-doping by atmospheric gases, such as water vapor, or be smaller than phase separated grains of PANI and PEO. However, thinner fibers are most suitable for sensor applications, smaller-diameter wires having a faster response associated with the more rapid diffusion of gas molecules through the wire. In the work by Liu *et al.* [109], a relatively high conductance of 0.5 S/cm was measured, again ascribed to PANI alignment during the electrospinning process.

Remarkably, the first work on blend-based fibers [25] reported one of the best performing results, namely PANI-PEO blend fibers with a conductivity of the mat of 0.1 S/cm, only slightly lower than the value measured on thin films, likely due to mat porosity.

### 3.4. Coaxial Electro-Spinning of Conducting Fibers

The coaxial spinneret setup has been rarely used in case of conducting polymers. Yu *et al.* [110] reported the formation of core-shell PANI-PVA fibers; however no conductivity data were provided. Recently, Zhang *et al.* [102] reported a comprehensive work where PANI fibers produced with different approaches are deeply characterized. Specifically, core-sheath PANI-PMMA structures are compared with fibers obtained from blend solutions, the former showing the best electrical conductance: after PMMA removal, a conductivity of 50 ± 30 S/cm has been achieved for a single fiber, that has been placed on interdigitated electrodes after electrospinning deposition, and pressed to optimize the contact. Conductivity is further enhanced to 130 ± 40 S/cm with a drawing, confirming the behavior highlighted by Pomfret *et al.* [103] in 2000, about the effect of mechanical treatments for polymer alignment. Conversely, mat conductivity is one order of magnitude lower, likely due to different fibers orientation, mat porosity or inter-fiber contact. Conductivity values obtained by Zhang *et al.* [102] are the best so far reported for conducting polymer fibers with diameters in the hundreds of nanometers to few micrometers range.

### 3.5. Template Synthesis

The method more often employed to process conducting polymers in fibrous morphologies is template synthesis: polymerization of aniline, EDOT and pyrrole onto fibers surface of different polymers (e.g., PMMA, Nylon) has been carried out. Although these systems do not always employ electrospinning, they are significant examples of development of conducting textiles. Template synthesis has been proposed for the deposition of conducting polymers onto yarns of fabrics, such as, cotton, wool, cellulose or silk [111–115]. In the case of polymerization onto electrospun fiber surface, suitable additives such as oxidizing salts or acids are required to promote the reaction and/or to get the external sheath doped. Such substances are added to the feed solution of the supporting polymer and critically affect the spinnability of the system, or can be dissolved in a reaction batch where the electrospun fibers are then dipped into. Following these approaches, conductivities up to 60 S/cm have been gained. This value refers to the vapor phase polymerization of EDOT on polyvinylpyrrolidone [76]. PS has also been used as the electrospun template for subsequent vapor phase polymerization of EDOT, affording a porous mat with a sheet electrical conductivity of around 1 S/cm [93]. EDOT polymerization in solution has been carried out on polyvinylchloride (PVC) mats, gaining a conductivity of 7.8 S/cm, together with significant mechanical and biocompatible properties [94]. Dong *et al.* [95] reported the *in-situ* polymerization of PANI onto preformed electrospun PMMA fibers, reaching a mat conductivity of 0.3 S/cm. As a general result, conductivity of PPy fibers obtained by template synthesis [89,92,116] is lower than the ones reported for PANI and PEDOT. The concurrent deposition of carbon nanotubes with PPy [117] led to an increase of conductivity, which however did not exceed 0.4 S/cm.

### 3.6. Functional Inorganic Fibers

Both the blend approach and the template synthesis, which have been discussed in the case of conjugated polymers, may also be applied to inorganic conducting materials.

Precursors added to the feed polymer solution and thus embedded in the resulting electrospun fibers can be converted by thermal activated reduction reactions in metallic materials. Conductivities as high as 104 S/cm have been obtained [118,119].

Metal coated nanofibers can be obtained by deposition of the conducting material onto an electrospun polymer template, which can be subsequently removed by thermal treatment or selective washing [120–122]. The method usually involves vacuum techniques (*i.e.*, metallization, electroless plating or sputtering) or specific environment, which limit the large scale production of fiber based devices.

Metallized electrospun fibers have been proposed for applications like electromagnetic shielding [123], or for the development of transparent electrodes. A remarkable result amongst the others concerns the deposition of both metals and transparent conductive oxides [124]: flexibility and possibility to transfer to different substrates have been demonstrated, together with a conductivity value as high as 2.2 × 10^5^ S/cm, comparable to bulk gold.

Yang *et al.* [125] demonstrated the encapsulation of Galistan liquid metal by means of coaxial electrospinning, working as electrode in light-emitting coaxial nanofibers; the second electrode, made of ITO, is obtained through evaporation directly on the fiber surface.

## 4. Semiconducting Polymer Fibers

Polymeric semiconductors are recently experiencing extensive interest because of the increasing enhancement of their electrical properties (e.g., charge carriers mobility) and the wide range of scalable processes that allow their deposition from solutions [9,10,126–132]. Despite generally thought for thin-film-based electronics, their formation within the cylindrical shape of the electro-spun fiber introduces many appealing aspects: a reduced amount of material consumption along with the intrinsic confinement of the active area, an increased surface-to-volume ratio for better functional interfacing (which is essential for sensing application and/or whenever anchoring sites are employed to enhance a specific property), improved flexibility and the possibility to finely pattern the active area just through the definition of the number and the direction of the nano-/micro-fibers employed within a device geometry; moreover, from a fundamental point of view, electro-spun nano-fibers may represent model systems for mono-dimensional charge transport study.

Driven by such motivations, many groups in the last decade have undertaken the challenge of the realization/implementation of electrospun semiconducting polymeric fibers, in general facing a lower solubility, and a lower molecular weight generally associated to highly conjugated molecular systems with respect to non-conjugated polymers. Nevertheless, efforts in the field have allowed not only to successfully overcome the above-mentioned processing issues, but also to explore the potential of electrospinning for investigating the relation between microstructure quality and anisotropy with charge transport properties.

In this section we aim at rationalizing the main steps which have conducted to the current level of advancement in this field, and to finally describe a possible scenario for further developments, with a special attention for the exploitation of such technology in logic circuits.

### 4.1. Mobility Extraction, Devices and Models

#### 4.1.1. Fiber Field Effect Transistors

**Figure 6.**
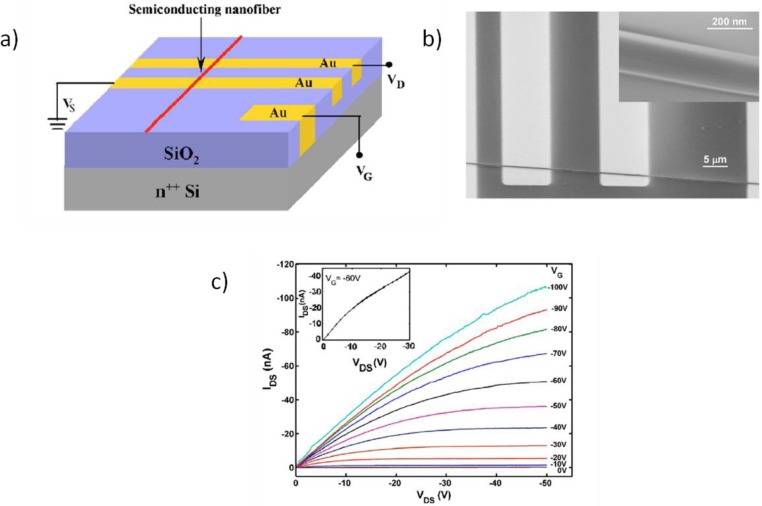
(**a**) Scheme of a Fiber Field Effect Transistor structure; (**b**) SEM image of a typical electrospun nanofiber deposited on pre-patterned SiO2 /Si substrate and (**c**) Ids vs. Vds characteristics of a fiber-Field Effect Transistor, showing accumulation mode operation when different negative gate bias are applied. Reprinted with permission from [133]. Copyright (2012) by AIP Publishing LLC.

One of the most common methods of investigation of the charge transport properties of semiconductors consists in their integration in Field Effect Transistor (FET) geometries; this enables the extraction of the “field effect mobility” (μ), which is a parameter describing charge transport characteristics in condition of high charge density. A FET (Figure 6) comprises two terminals (Source and Drain) in direct contact with the semiconductor and a third electrode (Gate) spaced from the semiconductor through a dielectric phase, representing a capacitive medium electrically isolating the Gate from the semiconductor [133]. When a potential difference between the Source and the Drain is applied (*V*_ds_ ≠ 0) just a low density of intrinsic mobile charges can flow through the semiconductor (*I*_ds_ ≈ 0) when no bias is applied to the Gate (*V*_g_ = 0); instead, when a positive (negative) Gate voltage is applied (*V*_g_ ≠ 0), a high density of negative (positive) mobile charges is induced at the dielectric/semiconductor interface, contributing to the semiconductor conductivity and producing an increase in the *I*_ds_ current. This is the typical operating condition of a polymer FET and it is indicated as accumulation mode. Only a few molecular layers at the interface with the dielectric are involved in the gate-induced charges accumulation [134] and consequently in the charge transport; importantly, the “channel” extension, *i.e.*, the extension of the area occupied by accumulated charges, and the charge density are only determined by the geometry of the capacitive coupling actually realized within the FET architecture, *i.e.*, by the specific capacitance (*C*_die_) of the device. Once defined *C*_die_, the following expressions valid for gradual channel approximation in a MOSFET [135] can be used for the extraction of field effect mobility values from the FET *I*_ds_ *vs. V*_g_ characteristic curves:

$$\mu =\frac{m_{\text{lin}}\times L^{2}}{V_{\text{ds}}\times C_{\text{die}}},V_{\text{ds}}\ll V_{g}$$ (2)

$$\mu =\frac{m_{\text{sat}}^{2}\times 2L^{2}}{C_{\text{die}}},V_{ds}≅V_{g}$$ (3)

where *m*_lin_ is the slope of *I*_ds_ *vs. V*_g_ plot, *m*_sat_ is the slope of *I*_ds_^1/2^ *vs. V*_g_ plot and *L* is the channel length of the FET.

#### 4.1.2. Capacitive Models

The most commonly used FET configuration for testing electruspun semiconducting fibers employs a planar geometry identical to that adopted for thin-films studies (Figure 6) [62,67,69,71,74,78,133,136–142]: fibers are transferred from the collector or directly deposited onto rigid substrates comprising highly doped silicon and silicon dioxide, which serve as (Bottom) Gate electrode and dielectric layer respectively. Gold electrodes either lithographically preformed onto the substrate or evaporated through a mask after the fiber deposition, serve as Source and Drain terminals. Considerable technological interest also lies in the realization of Top-Gate staggered fiber FETs, *i.e.*, FET geometries with the dielectric phase (and the gate electrode) located on the top of the fiber and the Source and Drain electrodes located below the fiber. However, fibers with diameters of hundreds of nanometers (which are the diameters usually realized by electrospinning) make such task quite challenging with standard polymeric dielectric layers, owing to the difficulties in realizing homogeneous films on the top of the fibers and due to the electrical resistance represented by the thick fiber bulk upon charge injection. As a consequence, so far ion gel dielectric phases have been preferred and successfully employed in top-gate configuration, as illustrated in the dedicated Section 4.1.3.

One of the main issues related to the extraction of the mobility of a semiconducting fiber in a planar FET geometry arises from the non-trivial estimation of the capacitance (*C*_die_) and of the charge density as a consequence. Given a specific geometry, the exact capacitance can be obtained as a solution of the Laplace Equation ∇^2^φ = 0 assuming a constant potential φ on the surface of the conductors/semiconductors. Well-established solutions are generally adopted as long as the ideal geometry is a good approximation of the actual geometry of the investigated device. While the simple case of parallel capacitors can be adopted for FETs based on thin film semiconductors [135], the capacitive model for a fiber on the top of a planar dielectric layer is not trivial: within the hypothesis of perfect cylindrical fibers, the contact between the fiber and the silicon dioxide dielectric plane is realized just through the tangential line between the fiber and the dielectric plane, out of which both the silicon dioxide and the air contribute in a complex way in defining the charge density profile along the fiber section.

**Figure 7.**
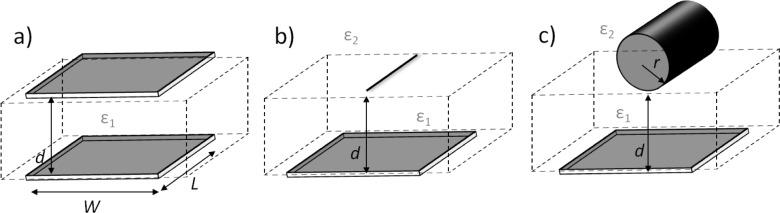
Capacitive models in use in fiber Field Effect Transistor (FET) mobility extraction procedures: (**a**) parallel plates capacitor; (**b**) wire-on-a-plane (*r* << *d*) capacitor and (**c**) cylinder-on-a-plane capacitor.

For parallel plate capacitors the following expression holds (Figure 7a):

$$C_{\text{die}}=\varepsilon_{0}\varepsilon_{r}\times \frac{A}{d}$$ (4)

where ε_0_ is the vacuum permittivity; ε_r_ the dielectric constant; *A* = *W* × *L* is the area of the overlap of the two plates and *d* is the distance between the plates. This has been rarely used for the extraction of electrospun fibers mobility, since it represents a satisfactory approximation just in the case of fiber radius much bigger than the dielectric layer thickness. When applied [67,78,136,138,139,141], the fiber was approximated to a thin film with channel width (*W*) equal to the fiber diameter and channel length (*L*) equal to the distance actually covered by the fiber between the Source and the Drain electrodes; in expression 4, the silicon dioxide thickness and dielectric constant values were used for *d* and ε_r_ respectively, actually neglecting any contribution on charge accumulation due to the presence of air between the silicon dioxide and the fiber.

Diversely, another well-known expression for *C*_die_ is the following [69,71,74,133,137,140,142] (Figure 7b):

$$C_{\text{die}}=\frac{2\pi \varepsilon_{0}\varepsilon_{r}\times L}{\ln(2d/r)}[143]$$ (5)

This expression is obtained as a solution of the Laplace equation for a conducting wire with radius *r* at a distance *d* from a conductive plane much bigger than the wire radius (*r* << *d*). This condition, plausible in the case of nanotubes based devices where the formula is originally taken [143], is actually never satisfied in the cases of the electrospun fibers reported in literature, where generally both the fiber diameters and the dielectric layer thicknesses measure hundreds of nanometers. More in details, expression 4 assumes that the whole fiber exposed surface is involved in the capacitive effect; this is not true in the common case of *r* ≈ *d* and leads to a systematic overestimation of the capacitance, which in turns results in an underestimation of μ values. Moreover, to account for the combined contribution of silicon dioxide and air to the overall capacitive effect, in all the reported cases the dielectric constant was approximated to the common value of 2.5, which is again unsatisfactory since it arises from the unrealistic assumption of a dimensionless wire within two infinite emi-spaces with ε_r_ = 1 and ε_r_ ≈ 4 (the dielectric constants of the air and the approximated one of silicon dioxide, respectively).

Alternatively, the exact expression for a cylinder-on-a-plane model has been recently used [62] (Figure 7c):

$$C_{\text{die}}=\frac{2\pi \varepsilon_{0}\varepsilon_{r}\times L}{{\sin\text{h}}^{-1}(d/r)}$$ (6)

Despite Equation (6) is representative of the actual geometry of a fiber of radius *r* distant *d* from a conductive plane irrespective of the radius of the cylinder, the evaluation of an equivalent dielectric constant (in the formula ε_r_) properly accounting for the relative capacitive contribution of air and silicon dioxide keeps being an unsolved issue. In the case of electrospun fiber FETs, the formula has been conservatively applied using ε_r_ = 3.9, which corresponds to the case of a wire immersed in a silicon dioxide medium rather than simply lying on a silicon dioxide plane, thus leading again to an overestimation of the capacitance value and an underestimation of the mobility.

#### 4.1.3. Ion-electrolyte Gated Devices

**Figure 8.**
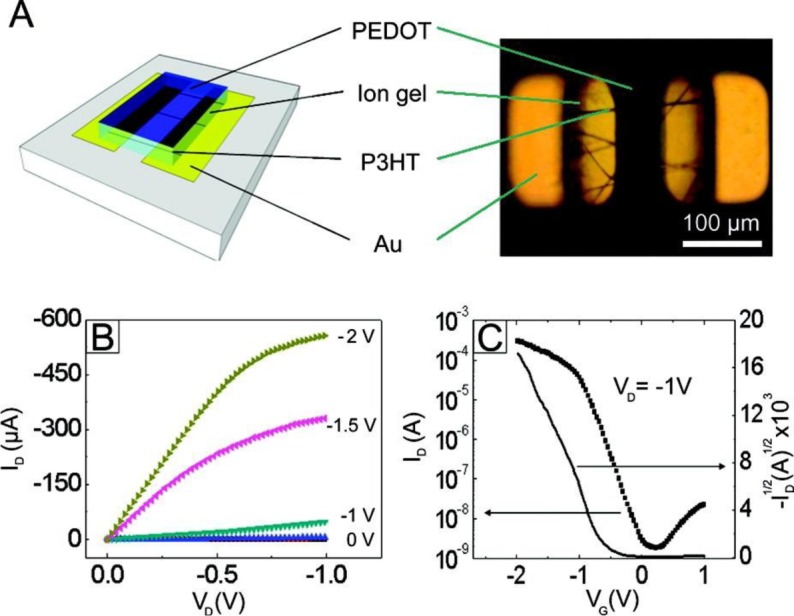
Example of device structure and optical microscopy of (**a**) an ion-gel fiber FET. In (**b**) and (**c**) the output and the transfer characteristic curves are reported, respectively Reprinted with permission from [148]. Copyright (2012) by American Chemical Society.

In order to induce a high charge carrier density in an electrospun semiconductor within a transistor geometry, the employment of ion-gels electrolytic dielectrics [144–147] has been proposed as a solution to better fit the geometry of the cylindrical wire (Figure 8) [62,148], thus benefiting of the whole fiber surface for the transport: upon biasing the electrolytic phase through a gate electrode, ions drift toward the semiconductor interface, leading to either the formation of an electrical double layer around the total interface, composed of ions on the electrolyte side and a high density of charges on the semiconductor side (field-effect regime), or to the occurring of electrochemical doping, in case of ions penetration through the semiconductor surface (electrochemical regime); in both cases the induced charge density is much higher than that induced by standard solid state dielectric layers. Within the ion-electrolyte gated configuration, an approach accounting for the *V*_g_ induced gate-displacement current [149] has been generally adopted for mobility extraction and, accordingly, the mobility values were obtained from the following Ohm’s law derived expression:

$$\mu =\left(\frac{L}{W}\right)\left(\frac{I_{d}}{pV_{d}}\right)$$ (7)

where the charge density *p* is calculated as:

$$p=\frac{Q}{eA}=\frac{\int I_{\text{disp}}dV_{g}}{\frac{dV_{g}}{dt}eA}$$ (8)

*I*_disp_ being the measured gate displacement current. In the ion-gel gating case the gate voltage *V*_g_ does not necessarily represent the electrochemical potential across the semiconductor-gel interface since at list the drop of potential at the gate/gel interface should be accounted for, in addition to drops of potential within the bulk of the electrolyte. The employment of a reference electrode inserted into the ion gel layer would actually allow for a more precise evaluation of the induced charge density, by measuring the potential difference between the reference electrode and the grounded Source electrode (*V*_ref_) and substituting it to *V*_g_ in Equation 8 [150].

Relatively high mobility values are often extracted from ion-gel based devices. Thanks to the induced high charge carrier density, electrolytic dielectrics can generally reveal improved transport in semi-crystalline polymeric semiconductors by filling the deep trap states otherwise strongly affecting the mobility [149]; however, the Coulombic interactions between the charges and the bulky ions of the electrolyte layer, possibly penetrated into the active phase [149], along with any other ion-induced phenomenon, do not allow a direct comparison between mobilities extracted from ion-gel gated and from standard ion-free dielectric gated devices.

### 4.2. p-Type and n-Type Fiber-FETs

In Table 2 (placed at the end of Section 4.2) we systematically report the most relevant cases of polymeric electrospun semiconducting fiber, indicating the specific electrospinning strategy employed, and the FET performance observed, in terms of field effect mobility (μ) and threshold voltage (*V*_th_). The previous overview on the different fibers based FETs geometry and the employed methods for mobility extraction must alert the reader that substantially different levels of approximation are present in the literature; moreover, in light of the nature of the gating model applied, a general tendency to mobility underestimation should be considered. In order to provide an effective tool of interpretation and comparison of the different performances published so far, in Table 2, besides reporting fiber materials, formation techniques and field effect mobility values, we have indicated fiber/dielectric geometries and dimensions, along with the gating model employed for mobility extraction.

It is clear from Table 2 that most of the investigations on p-type semiconductors processed by electrospinning and integrated in a FET have been carried out on regioregular poly(3-hexylthiophene) (rr-P3HT), a widely investigated semicrystalline polymer with well established charge transport properties [151–154]. The route that through the years has enabled reliable electrospinning of P3HT fibers with electrical characteristics comparable or even superior to those of P3HT thin films [62,71,74,133,137,144] is paradigmatic of the evolution of electrospinning techniques for polymeric semiconductors (as described in Section 2.4) and of their potential in terms of fine control of the polymer fibers microstructure, improved order, and distinct anisotropy. In the following, we separately account for the different electrospinning techniques and, where appropriate, the case of rr-P3HT is examined as an example to better define the relative advantages, constrains and limits of the various techniques.

#### 4.2.1. Single nozzle Electrospinning of Pure Semiconductors

**Figure 9.**
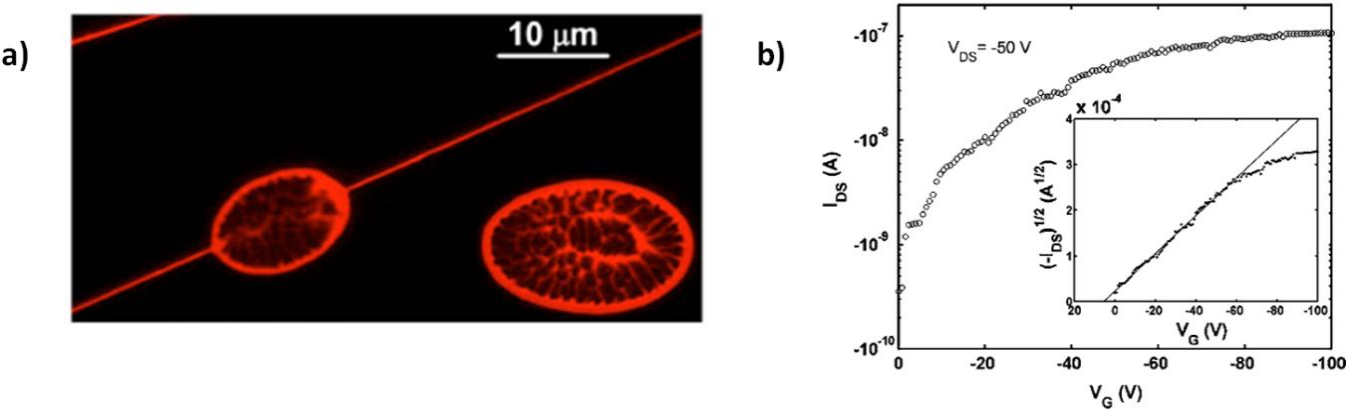
(**a**) Fluorescence image showing the morphology of droplets occasionally seen during single nozzle electrospinning of P3HT and (**b**) transfer characteristic curves for the same nanofiber FET operated at a constant drain bias of −50 V. Inset: curve fit of IDS1/2 vs. VG (channel length of 10 μm and fiber diameter of 180 nm). Adapted with permission from [133]. Copyright (2012) by AIP Publishing LLC.

The first attempts to realize electrospun P3HT fibers were carried out in 2005 by Liu *et al.* [133] and in the same year by Gonzales *et al.* [137] (Figure 9). They both simply dissolved the polymer in chloroform and employed a single nozzle, standard setup resulting in jet instability, diameter inhomogeneity and formation of beads and droplets, mainly due to the early evaporation of the volatile chloroform and solidification of the polymer already at the nozzle level. However, mobility as high as 3 × 10^−2^ cm^2^·V^−1^·s^−1^ was reported in Liu’s work, extracted from a single fiber FET with radius of 90 nm, using a planar device configuration with bare silicon dioxide as the dielectric layer. This value is one order of magnitude inferior with respect to the best P3HT thin films of literature [154]; the authors claim that oxygen and water exposure during the process may have contaminated the fibers and induced deep trap states for the charge carriers. However, the use of bare silicon dioxide with no passivation layer may have influenced the transport properties, since this was generally shown to be strongly detrimental of P3HT thin films electrical performances resulting in a drastic drop of mobility, down to values generally inferior to 1 × 10^−2^ cm^2^·V^−1^·s^−1^ [154]. In this scenario, the observed mobility is already surprisingly good considering the substrate employed and the intrinsic underestimation due to the capacitive approximation adopted, as commented before. The solvent employed also deserves further comments: the early evaporation at the nozzle may not only have induced uncontrolled spinnability, but also a small control of the crystalline rate and orientation within the fiber morphology.

**Figure 10.**
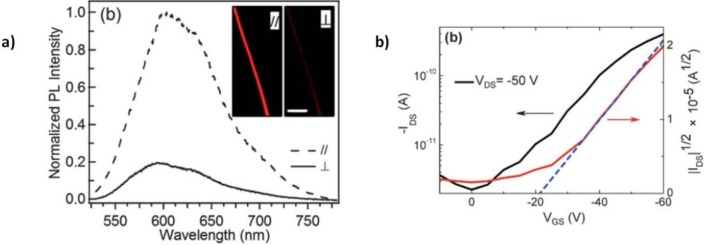
(**a**) Polarized photoluminescence (PL) spectroscopy of single MEH-PPV nanofiber showing clear molecular orientation along the fiber axis. PL spectra parallel to the fiber axis (PL//, dashed line) and perpendicular to the fiber axis (PL┴, continuous line) are reported. Insets: Corresponding fluorescence micrographs. Marker = 20 mm; (**b**) IDS (left vertical scale) and |IDS|1/2 (right scale) vs. VGS for VDS = 50 V of MEH-PPV nanofiber FET (channel length of 20 μm and fiber diameter of 500 nm). The dashed curve is a linear fit to data in the saturation region. Adapted with permission from [67]. Copyright (2010) by Royal Society of Chemistry.

On the basis of former experiences [68], in 2010 Deyu Tu *et al.* [67] applied single nozzle electrospinning to poly[2-methoxy-5-(2-ethylhexyloxy)-1,4-phenylenevinylene]) (MEHPPV) and firstly integrated the resulting fiber in a FET. MEHPPV is a light-emitting p-type polymeric semiconductor with intermediate charges mobility, which unlike P3HT displays an amorphous microstructure (Figure 10) [155–158]. The polymer, again dissolved in chloroform, is shaped in defect-free fibers, uniform and smooth as revealed by SEM and AFM, with diameters down to ~00 nm. Electrical performances comparable to the best thin films in literature were observed, with μ values of about 1 × 10^−3^ cm^2^·V^−1^·s^−1^; such a good mobility was associated to a strong molecular orientation in the fiber axis direction, as observed by Polarized transmission Fourier transform infrared (FTIR) spectroscopy, due to the elongational stress/strength induced by the high electric field during the electrospinning process.

#### 4.2.2. Coaxial Electrospinning: Pure Semiconductor and Composite Core-Sheath Fiber

In 2009, Lee *et al.* [74] largely improved P3HT fibers morphology by introducing a coaxial capillary spinneret as described in Section 2.5, here used to simultaneously feed pure solvent from the outer nozzle: the concentration of P3HT in the cone-jet at the inner nozzle tip is maintained low by providing chloroform from the outer nozzle, thereby preventing early solidification of P3HT. With this technique they obtained stable jets leading uniform and continuous P3HT fibers. However, on the corresponding single-fiber FETs on bare silicon dioxide they extracted a maximum field effect mobility of 1.7 × 10^−2^ cm^2^·V^−1^·s^−1^, which reflects the performances previously obtained with standard single-nozzle electrospinning setup [133,137]. Concordantly with such previous experiences, the lower mobility compared to state of the art P3HT thin film FETs was associated to air contamination during the process.

**Figure 11.**
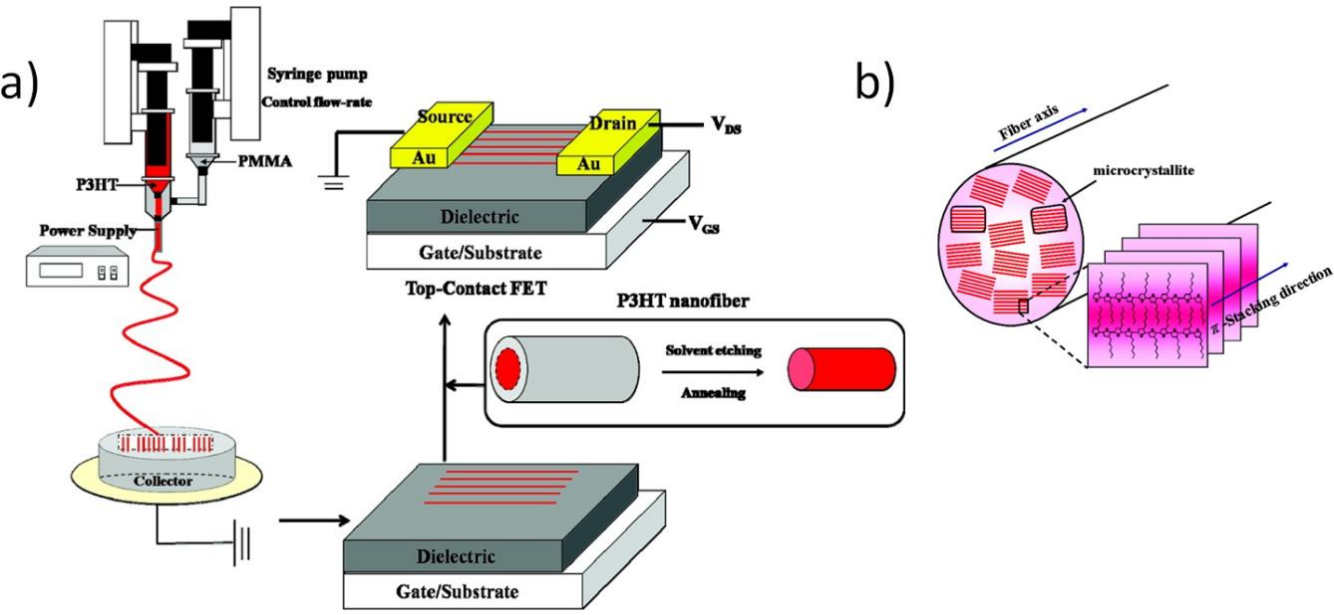
(**a**) Schematic representation of the coaxial electrospinning setup and process to fabricate the electrospun P3HT nanofiber based OFET; (**b**) schematic representation on the inner microstructure of single electrospun P3HT nanofiber. Adapted with permission from [71]. Copyright (2011) by American Chemical Society.

In 2011, Chen and *et al.* [71] improved P3HT fiber carrier mobility thanks to a modified coaxial setup, where the external nozzle was employed to simultaneously spin a polymeric solution (PMMA), as previously experimented on other polymeric semiconductors (Figure 11) [110]. This resulted in a core-sheath fiber structure, with the semiconductor embedded in a PMMA shell. With this technique, besides preventing direct air exposure of the semiconductor, they could also use a higher boiling point solvent for P3HT, with a consequent longer evaporation time, yielding increased crystallinity. FET performances were at least comparable to that of the best P3HT thin films (~ × 10^−1^ cm^2^·V^−1^·s^−1^), for a FET configuration in which this time silicon dioxide was treated with octadecyltrichlorosilane (ODTS) to obtain dielectric *passivation*, a method to isolate the active phase from the high trap-density silicon dioxide surface [154].

Further interest on the work of Chen *et al.* [71] lies in the deep investigation that they carried out on the morphology induced within the fiber by the electrospinning process. They used optical adsorption and Wide Angle X-Ray Diffraction (WAXRD) to check crystallinity and π–π interaction, and polarized photoluminescence to eventually detect a preferred molecular orientation, as it is expected by polymers when they undergo elongational stress. They observed two interesting phenomena: first, a totally peculiar morphology characterizes the fiber, in which crystalline domains are embedded in an amorphous matrix and are oriented with the π–π stacking parallel- and the molecules backbone perpendicular- to the fiber main axis. They also demonstrated to be able to control the degree of crystallinity and consequently the transport properties of the fiber just varying the shell flow rate of the PMMA solution; in fact, this results in a tunable shear stress applied to the semiconductor core during its solidification, which in turn affects the amount of crystalline aggregation. This again represents an impressive case of solid control of the functionality of a polymer through its morphology by electrospinning.

Such a strong anisotropy of π–π stacking, likely favoring the charge transport along P3HT fiber length, was further observed by the same group in another electrospun polythiophene derivative, poly{[2′,5″-5,5″′-di(2-ethylhexyl)-3′;5′,2″;4″,2″′]quaterthiophene-alt-3,6-dithien-2-yl-2,5-di(2-ethylhexyl)-pyrrolo[3,4-c]pyrrole-1,4-dione-5′,5″-diyl]} (P4TDPP) [140], in this case providing mobility much superior to that of the analogue thin-film device [159]. These observations, in addition to the numerous markers of strong molecular orientation observed in other polymeric semiconductors [62,67,70,77,78,160–162], indicates that electrospinning can be exploited as a general method to access active phases with controlled microstructural anisotropy, in contrast with films deposited by spin-coating in which planar morphological isotropy is generally encountered.

#### 4.2.3. Electrospinning of Polymeric Blends

**Figure 12.**
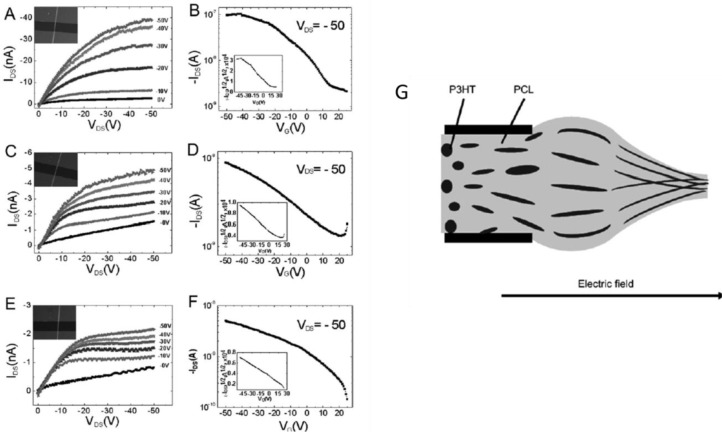
Output characteristics (IDS-VDS) at different gate voltages (VG) and transfer characteristics (IDS-VG) of (**A,B**) pure P3HT FET, and blend fiber FETs with (**C,D**) 20% PCL; (**E,F**) 50% PCL (channel length of 10 μm) and (**G**) schematic description of elongation of P3HT domains in highly concentrated PCL solutions under strong electric field and the formation of continuous P3HT fibrils in a PCL fiber. Adapted with permission from [74]. Copyright (2007) by Royal Society of Chemistry.

Electrospinning of polymeric blends is a general tool for tuning different properties of the fibers, like electrical and optical ones by using electroluminescent materials (MEHPPV) blended with high mobility semiconductors (P3HT) [136], and more often viscoelastic and electrical ones, by combining a polymeric semiconductor with an a high viscosity insulating polymer, mostly aimed at assisting the jet stabilization and the fiber formation. However, the presence of the insulating polymer has often been associated with a reduction of transport properties in the semiconducting phase within the fiber. In the work of Pinto and coworkers of 2003, doped PANI was blended with high viscosity PEO to realize the first reported case of electrospun p-type single-fiber FET; a limited mobility of ~ × 10^−3^ cm^2^·V^−1^·s^−1^ was measured and associated to the presence of non conducting PEO between polyaniline chains [69]. In the work of Lee *et al.* [74] P3HT/PCL blend low performances (~ × 10^−3^ cm^2^·V^−1^·s^−1^, even inferior when PCL ratio exceeding 30% is used) (Figure 12) are explained as an impeded formation of sufficiently big P3HT crystalline domains, but also impeded charge percolation due to defects within the semiconducting domains or traps at the interface between the semiconductor phase and the supporting polymer phase.

The various investigations of the microstructure of blended P3HT reported in literature provide a non-uniform picture in which, depending on the blending ratio, elongational strength and other electrospinning parameters, a more or less pronounced core-sheath micro-structured morphology is observed within the fibers, as a result of a more or less effective phase separation which is however rarely complete [62,70,74,77,136,160]. Interestingly, after the removal of the supporting polymer (PLC) with selective rinsing, Lee *et al.* [74] observed long and uniform nano-fibrils (diameter ~30 nm) touching each other, so that a continuous P3HT phase was formed and a uniform percolation path for the charge carrier guaranteed; this effect was claimed to depend on the different charges concentration under the effect of the electric field between the semiconductor and the supporting insulating material, leading to superior elongation of P3HT domains with respect to PEO during the field-induced process (Figure 12G).

In 2013, Chou *et al.* reported on the improvement of the performances of P3HT in blend with a semicrystalline polymeric insulating material (*i.e.*, poly(stearylacrylates), PSA) [142]; interestingly, they showed that a core-sheath structure is induced in electrospun blends, in which P3HT is protected by PSA side-chain crystallites that block the diffusion of oxygen and moisture from ambient. This largely enhanced ambient stability of P3HT-based electrospun nanofiber FETs. However, mobilities still inferior to that of pure P3HT were measured (μ_max_ = 3.2 × 10^−2^ cm^2^·V^−1^·s^−1^).

**Figure 13.**
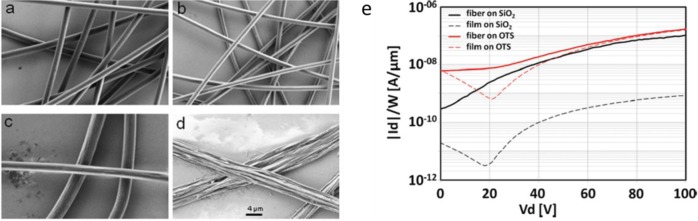
SEM images of P(NDI2OD-T2)/PEO fibers: the relative content of the two polymers is equal to (**a**) 70:30 mass/mass (w/w); (b) 70:30 w/w, upon rinsing with acetonitrile; (**c**) 50:50 w/w; (**d**) 50:50 w/w, upon rinsing with acetonitrile and (**e**) transfer curves of single fiber (continuous lines) and thin film (dashed lines) P(NDI2OD-T2) FETs (channel length of 20 μm); the characteristics of devices on both bare SiO_2_ (black lines) and OTS treated dielectric layers (red lines) are reported; a drain voltage of 100 V was applied during all the measurements; in the plot, drain current values are normalized to the channel width, which in the case of the fiber was conservatively assumed to be equal to its diameter. Adapted with permission from [78]. Copyright (2012) by American Chemical Society.

In 2012, Canesi *et al.* [78] adopted the supporting polymer strategy to realize the first reported n-type polymeric electrospun fiber (Figure 13). They employed a single nozzle setup and blended the popular n-type semiconductor poly{[ N,N ′-bis(2-octyldodecyl)-naphthalene-1,4,5,8-bis(dicarboximide)-2,6-diyl]-alt-5,5′-(2,2′-bithiophene)} (P(NDI2OD-T2)) [131,163,164] with the insulating PEO as the supporting material. Smooth and regular fibers with a circular-shaped cross section were obtained and, interestingly, fiber continuity and consistency was preserved even after PEO removal by selective rinsing, and occasionally long and uniform pure P(NDI2OD-T2) nano-fibrils touching each other similarly to previously mentioned Lee’s work [74] were obtained, as illustrated in Figure 13d, evidencing the effective phase separation between the supporting polymer and the semiconductor occurred during fiber solidification. A preferential orientation of the molecule with respect to the fiber axis was observed by using polarized infrared spectroscopy as a further confirmation of the preserved order in P(NDI2OD-T2) despite the presence of the PEO. As to the electrical characteristics, P(NDI2OD-T2) fibers formed in this way showed mobility at least similar to that of the corresponding thin film in the same device configuration. This was observed both before and after the removal of PEO, demonstrating that, due to phase separation, the presence of the supporting material in blend did not affect or perturbed transport within P(NDI2OD-T2) microstructure. Another interesting point of this work is that for the first time they investigated the effect of the substrate treatment on the transport properties of a single fiber FET. In fact, as well as P3HT, P(NDI2OD-T2) thin films performances are negatively affected by the use of bare silicon dioxide, requiring silicon dioxide *passivation* treatments. However, they showed that unlike the thin films, fibers transport properties are optimal even on the top of bare silicon dioxide, either in virtue of the small contact area realized between the semiconductor and the dielectric, or in virtue of the fact that the spun jet of P(NDI2OD-T2) solidifies into a fiber before getting into contact with the dielectric surface, so that interaction during the solidification stage is avoided.

### 4.3. Logic Circuits and Other Applications

We have so far emphasized the big effort done in demonstrating electrospun fibers with improved electrical characteristics for electronics application. Such interest has been lately accompanied by a realistic assessment of the necessary steps from lab-scale electrospinning process up to suitable scalable processes for possible industrialization [165]. Moreover, recently many works started focusing more on applications, with the aim of demonstrating for example that electrospun fibers can be effectively integrated in logic circuits with superior flexibility or, more ambitiously, embedded in fabrics or directly woven to form textile embedded logic elements— the so called “e-textile”.

While FETs, as those illustrated so far, represent a clear proof of concept for e-textile, since the FET is actually the building block of digital circuits, real applications deserve obviously more attention for what concern dimensionality, durability, reliability, pattern control, placing and manufacturability. Moreover, a critical aspect is represented by electrical contacting, *i.e.*, providing the fibers with suitable electrical connections compatible with a complex circuit routing.

**Figure 14.**
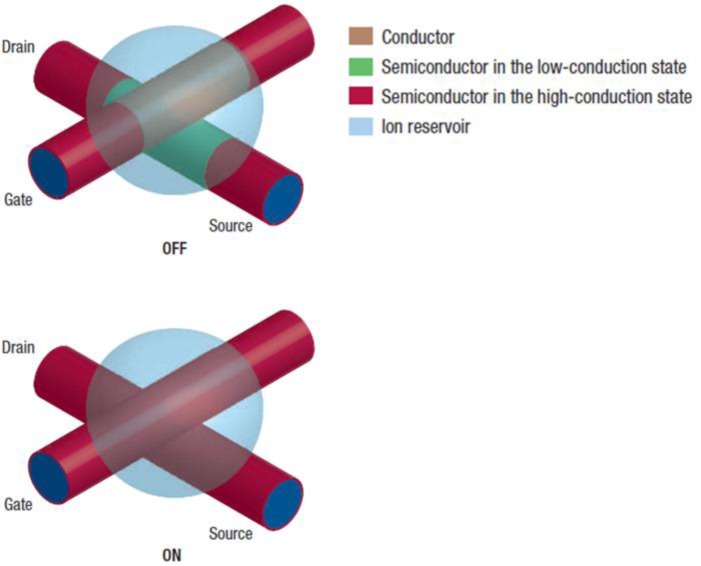
Schematic and working principle of an organic wire electrochemical transistor, formed at a fiber junction connected through an ionic liquid electrolyte. Adapted with permission from [168]. Copyright (2007) by Nature Publishing Group.

**Figure 15.**
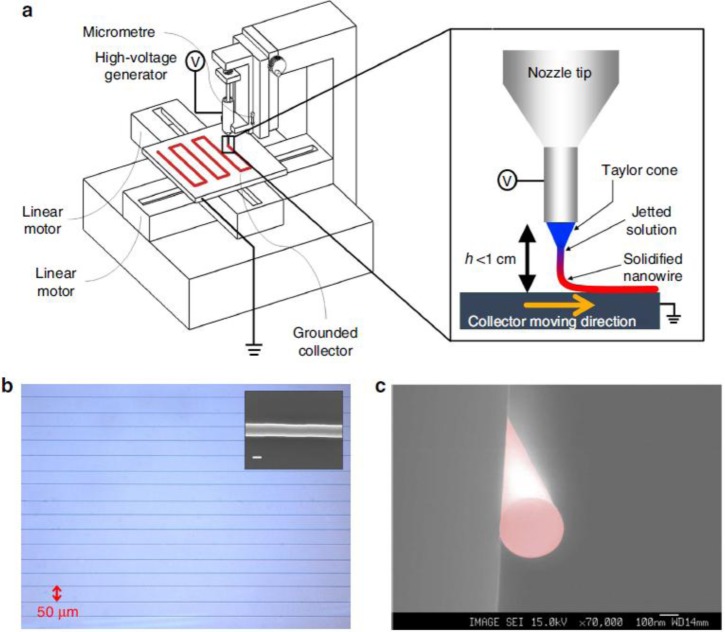
(**a**) Schematic diagram of organic nanowire (ONW) printer and nanowire (NW) printing process; (**b**) optical micrograph of well-aligned NWs (inset, scale bar, 200 nm) and (**c**) field emission scanning electron microscope image showing cross section of well-aligned NW, which forms a perfect circle. Reprinted with permission from [62]. Copyright (2013) by Nature Publishing Group.

Already in 2007 and 2009, woven logic obtained using non-electrospun organic conducting and semiconducting fibers was demonstrated by Hamedi *et al.* [166,167]. In both works, the structural key of the illustrated tri-dimensional micro-electronics is the use of an electrolyte dielectric phase (Figure 14) [168], which releases the FET geometry from the planar layered architecture and, as stated above, emphasizes fibers transport properties through an optimized dielectric coupling and a superior charge carrier density. In 2010, Lee *et al.* [144] gave a good demonstration of the advantages deriving from the employment of electrospun organic semiconductors, in terms of contact area with the dielectric layer, substrate compatibility, extreme flexibility, and easy scale-up (Figure 8). With the aid of ion gel electrolytic gate, they realized reliable arrays of electrospun P3HT OFETs, on flexible substrate (Polyethylene Terephthalate), working at operative voltage inferior to 2 V (which are values realistic for practical use) and with mobility values of ~ cm^2^·V^−1^·s^−1^. Just recently, a solid demonstration of large-area flexible electronics was provided by the work of Sung-Yong Min *et al.* [62] (2013); they proposed a setup for fast nanolithography based on the combination of the electrospinning working principle with a printing technology (Figure 15). With this setup they were able to align and pattern semiconducting nanowires with high speed, high precision and high reliability, achieving in ion-gel gated devices mobility up to ~0 cm^2^·V^−1^·s^−1^ and demonstrating working complementary circuit arrays (inverters) based on the patterning of both n-type (P(NDI2OD-T2)) and p-type (P3HT) nanofibers. They also observed P3HT/PEO microstructure, as obtained with their Nano-Wire Printer setup, and, differently from standard electrospinning, a well-established core-shell structure along the wire axis was found by TEM and elemental analysis, as an effect of a more effective phase separation.

We already mentioned in Section 3.5 a further possible application of electrospun fibers in the field of electronics, consisting in using them as template substrates for *in-situ* synthesis of conducting polymers. In this way, the electrospinning process is just used to realize the core-supporting phase obtaining the following advantages: decoupling the mechanical properties from the electrical properties, reduced morphological defectivity, improved molecular alignment along the fiber axis, easy core functionalization for electrical doping. This principle has been demonstrated to be successful in the realization of single-fiber FET based on doped PANI and PPy with unprecedented mobility, as high as ~1 cm^2^·V^−1^·s^−1^, which is a record value for standard planar ion-free dielectric layers [138,141]. The authors claim that such unrivaled electrical performances are mainly ascribable to two effects: (1) quasi-1D charge transport and reduced grain boundary effects and (2) Au nanoparticle on the top of PANI core [141] and highly doped PANI islands on the top of sulfonated poly(arylene ether ketone) (SPAEK) core [138] act as nano-electrodes, improving the transport by actually reducing the channel length of the FET.

**Figure 16.**
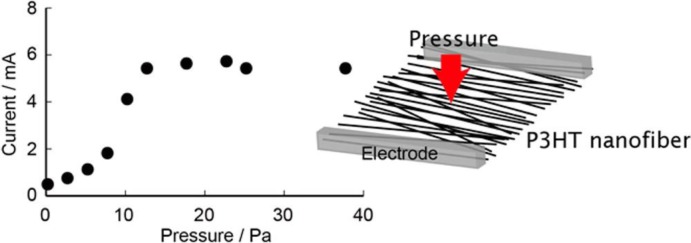
Schematic of the arrangement of the electrodes on the P3HT nanofiber assembly in the tactile sensor and current changes from P3HT nanofiber assemblies as a function of applied pressure. Reprinted with permission from [169]. Copyright (2012) by American Chemical Society.

To close the picture with a further widening of the possible range of exploitation of electrospinning process, we like to stress that interesting and smart applications of electrospun semiconductor fibers beyond the logic have also been thought and developed. Among these, simple tactile sensors based on aligned electrospun P3HT nanofibers have been developed for detecting small pressure changes and bending angles, as proposed by Qiang Gao *et al.* [169] in 2012 (Figure 16); single-fiber phototransistors and Light-Emitting Transistors (LIT) have been proposed by using electro-luminescent semiconducting materials [67,162]; finally, flexible and low-cost transistor memory devices based on hybrid P3HT: Au-nano-particles electrospun nanofibers have been recently published, exhibiting low operation voltages (± 5 V), large threshold voltage shifts (3.5–10.6 V), long retention ability (10^4^ s) and good stress endurance (100 cycles) [170].

## 5. Conclusions and Outlook

Electrospinning of conjugated polymers represents a powerful yet simple technique to form functional micro- and nano- fibers at low temperature, enabling both fundamental studies of the electronic properties of semiconducting polymers in confined dimensionalities and interesting opto-electronic and sensing applications. While fibers composed of only a single material are possible, given the additional difficulties in spinning conjugated polymers with respect to insulating ones, it is very common to form multi-component fibers where an insulating polymer enables the tuning of rheological properties for stable spinning. Interestingly, this is not necessarily limiting the performance of the active phase, and pristine fibers can be obtained with a post-deposition rinsing of the insulating phase. A series of techniques has been proposed in order to form multi-component fibers: spinning of blends from a single spinneret, multi-axial spinning of different materials and functional coating of supporting fibers.

In the case of conducting polymer fibers, interesting conductivities, in the range of 10–10^3^ S/cm have been achieved, especially thanks to the electro-spinning of PANI. The highest conductivity values can be achieved through a post-spinning mechanical drawing of the fibers. As far as semiconductors are concerned, we have focused here on charge transport properties. FETs are an ideal test structure for this purpose, besides being a necessary building block for logic applications. Both single fibers and multi-fibers/mats FETs have been demonstrated, and charge carriers mobility extracted with different models. Particular attention needs to be paid when extracting the carriers’ mobility owing to a non trivial estimation of the device geometry and of the specific dielectric capacitance in FET devices, an aspect which is not rarely overlooked in the literature. In the literature, several p-type fibers have been demonstrated, where P3HT has been the most employed polymer. Only recently, due to the recent development of high-mobility and stable electron transporting materials, n-type fibers have been demonstrated, offering the complementary unit for logic applications [171]. Charge carrier mobilities were demonstrated to equal the ones obtainable in highly uniform films deposited by spin-coating, while being relatively less affected by the particular substrate adopted, because of the on-the-fly solidification of the fiber. It can be easily foreseen that, thanks to the recent development of high performing donor-acceptor ambipolar copolymers, ambipolar fibers will be developed as well in the near future.

One of the great potentials of polymer fibers is their possibility to offer unconventional and new applications in the field of light detection, solar energy conversion, light emission and management, and electronic circuits. We have reviewed here in particular the application of fibers FET and interconnections in logic circuits, where the controlled patterning of quasi-1D structures could favor the development of high performance electronics. The integration of fibers within textiles could pave the way for large-area wearable electronics in an alternative and appealing way which would be followed by the integration of external plastic patches. In order to favor these applications, clear advancements are required in many aspects. The first one regards the controlled deposition and alignment of fibers with scalable processes. While electrospinning readily enables laboratory studies on processes parameters, materials and properties, in its most simple version it does not allow a simple method for patterning of circuital components. Clearly, this technique could gain a competitive edge with respect to inorganic wires and fibers, usually offering higher electronic performances, if transfer patterning or pick-and-place are avoided in favor of a single-step process where fibers are placed while formed. A series of options for advanced techniques have been proposed, comprising rotating jets and electrodes. One very promising method is represented by near-field deposition configurations, where the substrate is close enough to the jet to avoid the formation of a chaotic regime and to allow precise patterning. Complementary circuits based on fibers FET fabricated with this approach have indeed been demonstrated.

Another clear issue to be faced for the full deployment of the potentiality of fibers based electronics is an effective way of contacting the active phases and to interconnect different devices, especially in the directions of smart textiles. In this context, lithographically pre-patterned electrodes, which serve simple demonstrators, have to be replaced. The topology of circuits directly employing fiber conductors for electrodes and interconnections is not trivial and effective designs have to be devised. In perspective, the possibility to fabricate multi-component fibers could help this required development by the introduction of advanced and non-conventional device architectures. It is indeed worth noting that in multi-component fibers’ different functionalities may be integrated: the non-conjugated polymer, traditionally helping the stabilization of the jet, could also play an active role in providing, for example, a dielectric surrounding to a coaxially spun conjugated phase, which could be gated through it; electrodes could also be coaxial-formed [125,172], providing a possible solution for one of the most difficult tasks to be realized in the context of fibers based electronics. Circuit topology may be simplified by the use of electrolytes, which also serve as optimal gate media for the peculiar fiber aspect ratio.

It has to be kept in mind that polymer fibers share the same limits of organic electronics in terms of stability and reliability: the achievement of the necessary advancements in plastic electronics will also favor the actual application of organic fibers. One possible path to follow is represented by the integration of active polymer phases in synthetic fibers which are already in use for textiles, as demonstrated for example for liquid crystals incorporated in nylon fibers [17]. This approach may provide a certain level of protection at least to prevent degradation during processing and could envision the weaving of smart textiles if suitable layout strategies are developed.

## Figures and Tables

**Table 1.** Survey of conductivities achieved so far in polymer fibers.

Material	Deposition method	Fiber diameter	Tested sample	Conductivity (S/cm)	Year	References
PANI, doped with 2-acrylamido-2-methyl-1-propanesulfonic acid	Pure, from dichloroacetic acid, in coagulation solvent	220 μm	single fiber	150 1900 upon drawing	2000	[103]
PANI, doped with D,L-camphorsulfonic acid(HCSA)	Blend PEO, from chloroform	1.5 μm	mat	0.1	2000	[25]
PANI, doped with HCSA	(a) pure, from sulfuric acid; water cathode	(a) 139 nm	single fiber	(a) 0.1	2001	[105]
	(b) blend PEO 50 wt%, chloroform	(b) 419 nm		(b) 0.1		
	(c) blend PEO 72 wt%, chloroform	(c) 1320 nm		(c) 33		
PANI, doped with HCSA	blend PEO 10 wt%, chloroform	(a) 20 nm	single fiber	(a) 0.001	2003	[108]
		(b) 70 nm		(b) 0.01		
PANI, doped with HCSA	blend PEO 20 wt%, chloroform	120–300 nm	single fiber	0.01	2003	[69]
PANI, doped with HCSA	blend PEO 12.5 wt%, chloroform	180 nm	single fiber	0.5	2004	[109]
PANI, acid doped	pure, from formic acid, collected in acetone bath	100–1000 nm	single fiber	0.001–100	2007	[106]
PANI, acid doped	pure, from hot sulfuric acid in coagulation bath	(a) 100 μm	single fiber	(a) 1	2008	[104]
		(b) 370 nm in bundle		(b) 52.9		
PANI, doped with HCSA	(a) blend PEO	(a) from 1.2 to 2.7 μm	mat	(a) 8.1	2012	[102]
	(b) blend PMMA	(b) from 1.5 to 1.9 μm		(b) 0.01		
	(c) coaxial, PMMA shell, rinsed with isopropyl alcohol	(c) 620 nm		(c) 50 130 upon drawing		
PPy, doped with dodecylbenzene sulfonic acid	(a) pure, from chloroform	(a) 3000 nm	mat	(a) 0.5	2005	[100]
	(b) blend poly (vinyl cinnamate) 20 wt%	(b) ~000 nm		(b) 0.3		
PPy, doped with di(2-ethyl)sulfosuccinate salt	(a) pure PPy, in DMF	(a) 70 nm	mat	(a) 0.03	2006	[107]
	(b) blend PEO, in water	(b) 100–300 nm		(b) 0.0001		

**Table 2.** Survey of carriers mobility achieved so far in polymer fibers.

Material	Deposition method	Fiber diameter (nm)	Dielectric type and thickness	Capacitive model	μ (cm^2^·V^−1^·s^−1^)	*V*_th_ (V)	Year	References
PANI (p-type)	single nozzle + supporting polymer (PEO)	120	SiO_2_ 200 nm	(5)	1.4 × 10^−4^	82	2003	[69]
P3HT (p-type)	single nozzle	100 ÷ 500	SiO_2_ + HMDS 150 nm	(5)	3.0 × 10^−2^	5.5	2005	[133]
MEH-PPV/PHT (p-type)	coaxial with PVP solution	150 ÷ 300	SiO_2_ 300 nm	(4)	0.05 ÷ 1 × 10^−4^*	–	2005	[136]
P3HT (p-type)	single nozzle	670	SiO_2_ 300 nm	(5)	4.0 × 10^−4^	12	2005	[137]
P3HT (p-type)	coaxial with solvent (CHCl_3_)	500 ÷ 350	SiO_2_ 200 nm	(5)	1.7 × 10^−2^	12	2009	[74]
P3HT (p-type)	coaxial with solvent (CHCl3) + supporting polymer (PCL)	500	SiO_2_ 200 nm	(5)	1.2 × 10^−3^	16	2009	[74]
P3HT (p-type)	single nozzle + supporting polymer (PCL)	400	ion-gel	(8)	2.0	–	2009	[148]
MEH-PPV (p-type)	single nozzle	600	SiO_2_ + HMDS 100 nm + 300 nm	(4)	5.0 × 10^−3^	−22	2010	[67]
Au-doped PAN–PANI (p-type)	core: single nozzle (electrospun)	200	SiO_2_ 1200 nm + dry air	(4)	11.6	24	2011	[141]
	shell: gas phase polymerization							
Au-doped PAN–PPy (p-type)	core: single nozzle (electrospun)	200	SiO_2_ 1200 nm + dry air	(4)	1.2	−8.5	2011	[141]
	shell: gas phase polymerization							
SPEAK/PANI core/shell Nanofibers (p-type)	core: single nozzle (electrospun)	220	SiO_2_ 200 nm + dry air	(4)	3.0	−6	2011	[138]
	shell: liquid phase polymerization							
MEH-PPV (p-type)	single nozzle	500	SiO_2_ + HMDS 600 nm	(4)	9.4 × 10^−4^	8	2011	[139]
P3HT (p-type)	coaxial with PMMA solution	130	SiO_2_ + ODTS 200 nm	(5)	1.9 × 10^−1^	0.8	2011	[71]
P4TDPP (p-type)	coaxial with PMMA solution	194	SiO_2_ + ODTS 200 nm	(5)	3.0 × 10^−1^	−1.25	2011	[140]
P(NDI2OD-T2) (n-type)	single nozzle + supporting polymer (PEO)	1180	SiO_2_/SiO_2_ + OTS 230 nm	(4)	9.0 × 10^−2^	−1	2012	[78]
P3HT (p-type)	single nozzle + supporting polymer (PSA/PnLA)	181	SiO_2_ + ODTS 300 nm	(5)	3.2 × 10^−2^	4.18	2013	[142]
P3HT (p-type)	single nozzle** + supporting polymer (PEO)	780	SiO_2_ 100 nm	(6)	3.0 × 10^−2^	−1.5	2013	[62]
P(NDI2OD-T2) (n-type)	single nozzle** + supporting polymer (PEO)	248	SiO_2_ 100 nm	(6)	1.2 × 10^−2^	–	2013	[62]
P3HT (p-type)	single nozzle** + supporting polymer (PEO)	780	ion gel	(8)	3.8	–	2013	[62]

* Extracted from web. The effective field-effect mobility of holes in these blend nanofibers are 1 order of magnitude higher, if the fact that the web of nanofibers occupy only 10% of the FET channel area is taken into account;

** fibers fabricated using organic nano-wire printer.
